# Left Atrial Myxoma Following Coronary Artery Bypass Grafting with Patient Coronary Arterial Grafts: a Rarity

**DOI:** 10.21470/1678-9741-2016-0080

**Published:** 2017

**Authors:** Kartik Patel, Kumar Rahul, Malkesh Tarsaria, Amber Malhotra

**Affiliations:** 1 Department of Cardiovascular and Thoracic Surgery of U. N. Mehta Institute of Cardiology and Research Center (affiliated to BJ Medical College, Ahmedabad), Gujarat, India.

**Keywords:** Myxoma, Coronary Artery Bypass, Heart Atria

## Abstract

The development of left atrial myxoma after coronary artery bypass graft surgery is a rare entity. A 60-year-old man with previous off-pump coronary artery bypass grafting four years ago with patent coronary grafts was diagnosed with left atrial mass. The patient underwent successful resection of the same through minimally invasive right anterolateral thoracotomy. Histopathology of the atrial mass confirmed the diagnosis of atrial myxoma.

**Table:** 

Abbreviations, acronyms & symbols	
TEE	= Transoesophageal echocardiography

## INTRODUCTION

Atrial myxomas are the most common primary cardiac tumors. Cardiac myxomas represent 30-50% of all benign tumours. About 75% originating from the left atrium and 15-20% from the right atrium, near the interatrial septum at fossa ovalis, and occur in all age groups. If left untreated, they progressively enlarge and are potentially fatal^[1,2]^. We herein report a patient with left atrial mass attached to posterior wall of left atrium, who underwent coronary artery bypass grafting four years ago with patent coronary artery bypass grafts.

## CASE REPORT

**Fig. 1:**
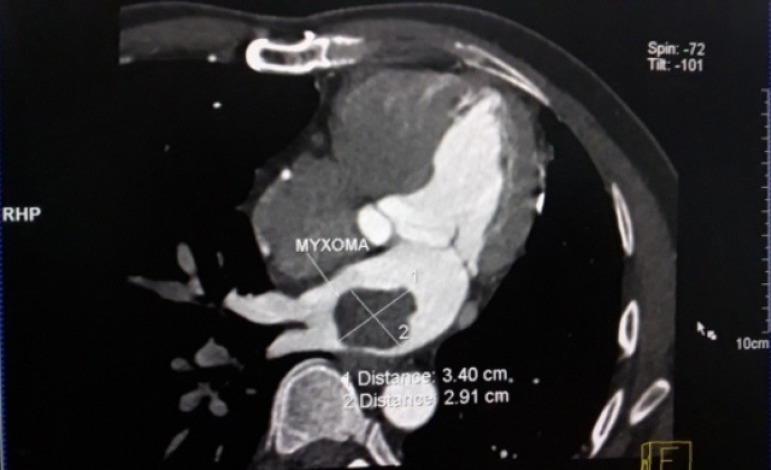
Computed tomography scan showing large left atrial intracardiac mass.

A 60-year-old man was admitted with complaints of dyspnea grade II and single episode of syncope. The patient previously underwent coronary artery bypass grafting four years ago. On investigation, chest X-ray and electrocardiogram were unremarkable. Transthoracic echocardiogram revealed large (4.20 x 3.50 cm), mobile and pedunculated mass attached to posterior wall of left atrium with no obstruction of mitral valve orifice. Biventricular function on echocardiography appeared normal, with no other significant pathology. Computed tomography scan confirmed presence of large, lobulated and hypodense mass in left atrium, measuring approximately 4.00 x 3.50 cm attached to posterior wall. Coronary artery bypass grafts were patent without any evidence of stenosis or occlusion (Figure 1).

**Fig. 2:**
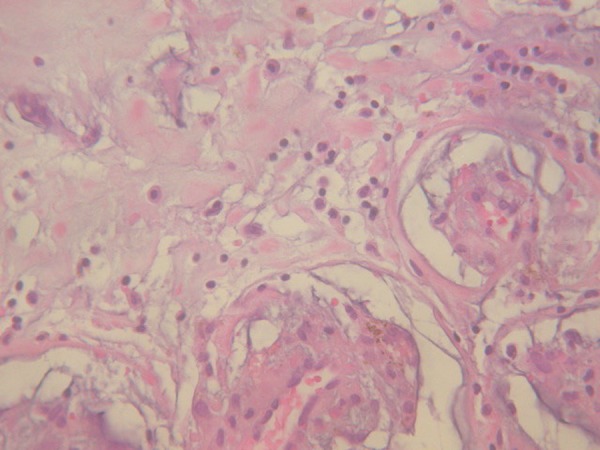
Histopathology of resected specimen of left atrial mass suggestive of myxoma.

Patient underwent reoperation through right anterolateral thoracotomy in 4^th^ intercostal space. Cardiopulmonary bypass was instituted through femoro-femoral cannulation. Temperature was lowered down to 22ºC and heart was fibrillated. Left atrium was opened without aortic cross-clamp and mass resected *in toto*. Post-resection thorough saline wash given and posterior wall cauterized to prevent recurrence. Left atrium closed in two layers. Patient was successfully weaned off from cardiopulmonary bypass. Total fibrillatory arrest time was 25 minutes and cardiopulmonary bypass time was 95 minutes. Histopathological examination of the mass confirmed the diagnosis of myxoma (Figure 2). We continued with follow-up of the patient by echocardiography right after surgical treatment, as well as after 3 months and after 6 months, with no signs of tumor recurrence.

## DISCUSSION

Left atrial myxoma developing late following coronary artery bypass grafting with patent coronary grafts have not been frequently reported in literature. Yavuz et al.^[3]^ reported resection of left atrial myxoma with concomitant redo coronary artery bypass grafting through median sternotomy approach. In their case, the patient underwent coronary artery bypass grafting eleven years ago. Kollias et al.^[4]^ reported resection of right atrial appendage myxoma through median sternotomy with patent coronary grafts following urgent coronary artery bypass grafting six months ago due to left main disease. Mishra et al.^[5]^, in their large single-centre experience with intracardiac myxomas, studied the clinical presentation and recurrence rate of cardiac myxomas and reported successful resection of intracardiac myxomas through median sternotomy approach. In their study, occurrence of intracardiac myxoma following coronary artery bypass grafting was not reported^[5]^. The association of coronary artery disease with left atrial myxoma has been reported earlier, but development of left atrial myxoma after coronary artery bypass grafting with patent coronary grafts is a rare entity.

In our case, the patient had undergone coronary artery bypass grafting using left internal mammary artery graft to left anterior descending. Left radial artery to obtuse marginal branch and saphenous vein graft to posterior left ventricular branch of right coronary artery through median sternotomy. Left atrial mass was detected by two-dimensional echocardiography and transoesophageal echocardiography (TEE). Preoperative computed tomographic angiography scan was performed to confirm the diagnosis and evaluate graft patency. On computed tomographic angiography scan all the coronary grafts were flowing well. Right coronary artery graft was lying retrosternally in right atrioventricular groove making it prone for injury during sternotomy. We preferred minimally invasive right thoracotomy approach over routine median sternotomy to avoid graft injury and extensive dissections due to presence of patent grafts. Fibrillatory arrest avoids the need for aortic cross-clamping and cooling allows flow reduction for brief periods intermittently. Following surgery, histopathology of the mass confirmed myxoma.

The surgical approach of left atrial myxoma with previous coronary artery bypass grafting is an important issue from the viewpoint of preventing injury to patent coronary grafts, intraoperative embolization of myxoma and myocardial protection. All patients must undergo computed tomographic angiography scan to look for graft patency. Patients with patent coronary grafts should undergo minimally invasive/right thoracotomy approach whereas in cases where coronary grafts are not patent then median sternotomy with concomitant coronary bypass grafting and resection of myxoma should be performed.

## CONCLUSION

In conclusion, occurrence of left atrial myxoma following coronary artery bypass grafting is a rare entity. Resection of the same with patent grafts can be safely tackled by minimally invasive approach under fibrillatory arrest with good outcome.

**Table:** 

Authors' roles & responsibilities	
KP	Substantial contributions to the conception or design of the work; drafting the work or revising it critically for important intellectual content; final approval of the version to be published
KR	Substantial contributions to the conception or design of the work; drafting the work or revising it critically for important intellectual content; final approval of the version to be published
MT	Substantial contributions to the conception or design of the work; drafting the work or revising it critically for important intellectual content; final approval of the version to be published
AM	Substantial contributions to the conception or design of the work; drafting the work or revising it critically for important intellectual content; final approval of the version to be published
